# Parent Perspectives on Water Safety for Children with Autism

**DOI:** 10.1007/s10803-025-06819-7

**Published:** 2025-04-16

**Authors:** Barbara D. Cosart, Karla A. Lawson, Stewart R. Williams, Kayla Elisabeth Lewis, Rashidah Namutebi, Molly B. Johnson

**Affiliations:** 1 Trauma Services, Dell Children’s Medical Center, Austin, TX USA; 2 Trauma and Injury Research Center, Dell Children’s Medical Center, Austin, TX USA; 3 Department of Surgery and Perioperative Care, Dell Medical School, University of Texas at Austin, Austin, TX USA; 4 School of Health Sciences, Regis College, Weston, MA USA; 5 Kinesiology Department, University of the Incarnate Word, San Antonio, TX USA

**Keywords:** Water safety, Drowning, Autism, Swimming lessons

## Abstract

Among children with autism, drowning is the leading cause of death through 14 years old. Attributes such as wandering, sensory issues, impulsivity, and a limited sense of danger could increase risk. In addition, autistic children often have trouble participating in structured group activities like swimming lessons. The purpose of this study is to better understand water safety experiences of parents of children with autism. Parents of autistic children were asked to participate in a focus group about their family’s water safety experiences. Six focus groups and one interview were held. Six major themes were identified from transcripts that were common among participants: autism characteristics influence water safety risk; water safety fears influence family life; it is difficult to find and access water safety and swimming lesson information for children with autism; autism characteristics affect participation in swimming lessons and other aquatic activities; autistic children have unique swimming lesson needs; and instructor preparedness is key to swimming lesson success for autistic children. Water safety needs to be elevated in importance for families of autistic children. Information on drowning risk and prevention should be made more accessible. Barriers to adaptive swimming lessons such as cost, scheduling, and availability need to be addressed. More training opportunities need to be provided to swimming instructors to improve teaching children with complex needs, such as those with autism.

**Supplementary Information:**

The online version contains supplementary material available at 10.1007/s10803-025-06819-7.

Drowning is a major public health issue worldwide (World Health Organization, 2024). According to the Centers for Disease Control and Prevention (Centers for Disease Control and Prevention [CDC], 2024), in the U.S., drowning takes the lives of about 4,000 people per year. Additionally, drowning is the leading cause of death in children 1–4 years old and a leading cause of death for children of all ages. Drowning is not always fatal, though; many people are treated for injuries resulting from nonfatal drowning (CDC, 2024). Drowning injury can have long-lasting, devastating effects, with many people who experience a drowning injury suffering irreversible brain damage due to oxygen deprivation (Suominen et al., 2014). Without many treatment options, drowning prevention is the best mechanism for mitigating drowning-related injury. In order to focus drowning prevention efforts most effectively, it is critical to understand populations at increased risk for drowning.

Children and adolescents diagnosed with autism are at particularly high risk of drowning. Among children with autism, drowning remains the leading cause of death through 14 years of age (Guan & Li, 2017). Autistic children and adolescents are three times more likely to drown than their neurotypical peers (Peden & Willcox-Pidgeon, 2020). Deaths for autistic children are nearly 40 times as likely to be caused by drowning as for the general population (Guan & Li, 2017). With more than one in thirty children aged 3–17 years diagnosed with autism (QuickStats, 2024), there is a large number of children and adolescents for whom drowning prevention should be a top priority.

Characteristics and behaviors common with autism may increase the risk of drowning. Many autistic children are prone to wandering or elopement, which may contribute to the higher risk of drowning when near bodies of water (Rice et al., 2016; Guan & Li, 2017). Additionally, poor awareness of safety risks can make drowning and other injuries more likely (Pardej & Mayes, 2024). Autism characteristics can also make it less likely children and adolescents will learn water safety and swimming skills necessary to keep them safe around water. Up to 97% of autistic children have atypical sensory processing (Dellapiazza et al., 2018), which can mean that swimming pool environments pose sensory challenges (e.g. loud, bright, or cold). Behaviors such as resistance to transitions, can lead to safety issues in the pool environment (Dowdy & Tincani, 2020). These individual barriers may be associated with historically poor access to water safety resources for the autistic community.

Structural barriers to participating in water competency and swimming lessons also exist for children with special needs. Autistic children may be limited by a shortage of swimming instructors with appropriate experience and by the increased cost of personalized instruction (Ananthapavan et al., 2024). Furthermore, swimming instructors report a need for additional preparation and support in teaching children with autism (Carter & Koch, 2023).

Despite the many barriers to participation in water safety education, swimming lessons can have profound benefits for autistic children, with impacts on many aspects of their life and behavior. Swimming lessons with instructors skilled in working with the autistic population have been shown to successfully improve swimming skills (Alaniz et al., 2017; Caputo et al., 2018; Fragala-Pinkham et al., 2011; Johnson et al., 2021; Munn et al., 2021; Pan, 2010; Vodakova et al., 2022), with improvements maintained after 6-months (Zanobini & Solari, 2019). Other physical improvements have included locomotor skills, gross motor skills, balance, and sleep habits (Battaglia et al., 2019; Marzouki et al., 2022; Ansari et al., 2021a, b; Mische Lawson & Little, 2017). Additionally, swimming lessons have been shown to impact mental/emotional factors, such as reducing emotional reactivity and improving adaptation to change, relational skills, social skills, and psychosocial health (Caputo et al., 2018; Johnson et al., 2021; Pan, 2010; Zanobini & Solari, 2019; Güeita-Rodriguez et al., 2021). Research has shown swimming lesson programs to be feasible, acceptable, and effective and to reduce parent stress [Bekhet et al., 2023; Kemp et al., 2023).

The purpose of this study is to better understand water safety experiences of parents of children with autism, regardless of the child’s functional level, swimming ability or swimming lesson experience. In particular, we wanted to identify specific challenges that parents face in being in water environments, factors that parents believe increase their child’s risk, and barriers and facilitators to successfully participating in swimming lessons.

## Methods

This research used focus groups to gather qualitative data on the lived water safety experiences of parents of autistic children. An ecological model of health behavior and the understanding that behavior is shaped by the interplay of multiple influences, guided the development of this project and thematic analysis (Sallis & Owen, 2015). Additionally, a brief survey gathered demographic and background data on the parents and children.

### Procedure

Potential participants were recruited from social media posts, autism and special needs email groups, and therapy clinics in central Texas to participate in a focus group about their water safety and swimming lesson experiences. Participants were adults who self-identified as parents of an autistic child (0–17 years old) and were residing in the Austin, Texas metropolitan area. Interested parents completed an online screening questionnaire verifying eligibility, and were contacted by email or phone to schedule attendance at a focus group.

Four focus groups took place at a hospital conference room or library in different parts of the metropolitan area. Two focus groups were held online. One parent who could not attend a focus group was interviewed online. Focus groups had 2–6 participants. Group or interview discussions were 60–90 min long and moderated by authors BDC and MBJ. Focus group discussions were guided by the questions in Table 1. Focus groups and interviews were audio recorded. Information on participant demographics and the swimming lesson experience of their children were collected in a brief online survey at the beginning of each focus group or interview.

Screening and participant surveys were managed using REDCap (Research Electronic Data Capture) electronic data capture tools (Harris et al., 2009, 2019). Institutional Review Board approval for the research was granted by the University of Texas at Austin Health Sciences Institutional Review Board (STUDY00004734).

### Data Analysis

Transcripts of the focus group and interview discussions were generated by Google Meet, which was used to record both online and in-person sessions. Each transcript was carefully revised and edited for accuracy by one member of the study team (BDC) using audio recordings from Google Meet and a backup audio recorder for reference. Both deductive and inductive approaches were used to identify relevant concept codes (Saldaña, 2021). Coding was conducted within an experiential qualitative framework, in order to capture rich meaning of participants’ lived experiences (Braun & Clarke, 2021). Prior literature reviews as well as authors’ lived experiences with autism or with autistic family members contributed to deductive identification of initial codes, which were revised inductively as each group or interview was conducted. Codes, both latent and semantic, were drafted and revised using Reflexive Thematic Analysis, iteratively assessing how codes were used to capture participants’ lived experiences (Braun & Clarke, 2021). Coding reliability was not measured, as the goal was to identify rich, nuanced meanings rather than measure the prevalence of concepts, which cannot be accurately captured with this data collection method. The coding process was led by one author (BDC), with refinements suggested by two other authors (KEL, RN). The lead coder (BDC) led the thematic analysis with the help of a fourth author (MBJ).

**Table 1:** Water safety-related focus group questions for parents of autistic children

What are some ways you have heard about to keep kids safer around water? What information has been particularly helpful or not helpful?
Where did the water safety information you heard about come from (e.g. doctor’s office, school, parenting books, therapy offices, media, personal stories or communications, etc.)?
In your experience, what are some ways that autism has made water safety efforts more challenging than they might have been?
Describe your experience with challenges in the home (such as with bathtubs, toilets), and those outside (pools, ponds, buckets).
How does your child’s autism shape choices you make about using safety devices, like life jackets?
How have water safety concerns shaped recreational or vacation plans, such as where or how often you take them swimming or where you travel?
What personality traits or attributes of your child might affect their water safety risk?
Let’s talk about times when you felt your child had or could have had a close call with drowning. Describe the situation and environment. Was there a time where nothing happened, but you realized it could have?
Let’s talk about times when your child was enrolled in or attending swim lessons: Describe your experience with your child taking swimming lessons. What made a swimming lesson experience difficult for your child? What helped make your child’s swimming lesson experience easier or successful?
If you could have created the perfect swimming lesson experience, what might have helped to make swimming lessons easier or successful for your child?
What are the skills you are or were most interested in your child developing during swim lessons? What made it easier or harder for them to develop these skills?
Did your water safety and swimming skill expectations for your child change as they got older?
Let’s talk about times when you tried to find or enroll your child in swim lessons: Describe your experience looking for swimming lessons for your kid with autism. What made your experience hard? Were there things that helped make your experience easier?
Let’s talk about other younger or older children, if you have them. How has having a sibling with autism impacted their water safety or swimming experiences?
Is there anything else about your water safety or swimming lesson experience related to autism that you think is important to share?

## Results

### Participants

A total of 21 adults participated in this study (95% female, 5% male). The average age of the participants was 44 years (range 32–58 years). Participants were parents of 1–3 children with autism; participants reported on the experiences of 25 autistic children (72% male, 20% female, 8% Trans/non-binary). The average age of the autistic child was 11 years (range 3–18 years).

### Swimming Lesson Experience

In the preliminary survey, 42.9% of parents reported that their autistic child had completed multiple years of swimming lessons; 33.3% reported completion of multiple sessions of swimming lessons; 14.3% reported that their child had attempted, but not completed more than one session of swimming lessons; and 9.5% reported that their child had never attempted swimming lessons.

### Water Safety Themes

Six major themes were identified from focus group and interview transcripts that were common among participants (Table 2). Participant quotes are identified using roman numerals for the focus group and arabic numbers for the participant number. Additional quotes are available in supplementary Table 3.

**Table 2:** Water safety themes and subthemes identified from focus groups of parents of autistic children

**Theme 1: Characteristics of autism influence water safety risk**
Being drawn to water
Poor sense of danger
Wandering and elopement
Impulsivity
Opposition
Communication difficulties
**Theme 2: Water safety fears influence family life**
Emotional toll of vigilance and planning
Water avoidance
Water access in travel planning
**Theme 3: Water safety and swimming lesson information for autistic children difficult to find or access**
Insufficient water safety information
Difficulty identifying specialized swimming lessons
Limited availability of specialized or adaptive lessons
Distance to specialized lessons and higher costs
Competing priorities
Racial and ethnic disparities
Prior negative swimming instruction experiences
**Theme 4: Characteristics of autism affect participation in swimming lessons and other aquatic activities**
Preparing for water activities and transitions
Sensory overload in pool environments
Water can be a negative sensory stimulus
Strong responses to water recreation gear
Water can have a positive sensory effect
Difficulty self-assessing sensations
Masking
Inflexibility, fixations, and obsessive rule-following
Other diagnoses and physical challenges
**Theme 5: Autistic children have unique swimming lesson needs**
Communication and learning challenges
Developmentally appropriate
Learn by observing peers
Dedicated learning environment
Parent involvement
**Theme 6: Instructor preparedness is key to swimming lesson success for autistic children**
Experience teaching individuals with autism
Knowledgeable about autism
Therapist skills

### Theme 1: Characteristics of Autism Influence Water Safety Risk

Parents reported that many common characteristics of autistic children may put them at greater drowning risk when they are in or around water. A prominent theme throughout the conversations was that many autistic children are *drawn to water*. One parent described her daughter’s “deep and abiding fascination with deep water”(II,45). This attraction to water can lead children to access water unexpectedly. A parent described her son, “He sees water and he wants to just run and jump [in]” (I,30). Several parents also noted their autistic children have a *poor sense of danger*. One parent noted, “He feels like he can do it… [I] want to nurture that confidence and that independence…but he doesn’t understand yet why something is inherently dangerous” (VI,35). Most parents reported that their autistic children may *wander or elope* from a space they are expected to be in. “My son has a habit of eloping, like he’s so *impulsive*. I can’t just stand or tell him to walk…I have to hold his hand” (I,30). Some parents pointed out that their children with autism can be very resistant to protective measures or have a tendency towards *opposition*. One parent explained that her son “will not do things on demand; [there is] a subgroup of kids with autism that have that kind of defiant thread…he won’t get upset about it. He just won’t do it and he totally understands and he’ll do it another time. Just not upon request” (IV,43). *Difficulties with communication* and learning can also affect water safety. One parent recalled a very stressful moment when her child eloped near water, and she worried that his communication deficits might hinder his ability to find help. “I found him, but it was very scary because…he communicates, but people might not understand him” (I,30).

### Theme 2: Water Safety Fears Influence Family Life

Parents reported experiencing a tremendous *emotional toll from the vigilance and planning* associated with their autistic child’s complex needs. “It is incredibly isolating and it impacts every minute of every day…you’re exhausted from coordinating all the services that you have to do” (IV,43). Another parent reported how water activities require a lot of planning, “If you forget something like…the right goggles…the snack you always bring, it can all go south…[being] constantly vigilant is rough” (II,45).

Some parents pointed out that the high level of vigilance needed to keep autistic children comfortable and safe can lead parents to *avoid being around water*. “I used to take my son [to watch his sister on the swim team] but I’m scared. I don’t want to take him because he used to run and he will…jump in the water” (I,30). Another parent reported, “I don’t really go to my brother’s house if they’re gonna have a get-together because there’s a swimming pool…they’re leaving the gate open…they can just tell their kids to…stay out…[but with him] it doesn’t work that way” (IV,43).

Many parents reported *considering water access in travel planning*, with most avoiding being near water for safety reasons. One parent shared they “tend to do Airbnb or a house when we travel and…no pools at all” (V,8). Another family will “avoid going to places with water” (I,27). On the other hand, another parent pointed out that his son feels such a need to be in the water that if they are away from home, they plan around access to a pool. “If we come too late [to the hotel] and the swimming pool is already closed, he might have a breakdown” (V,10).

### Theme 3: Water Safety and Swimming Lesson Information for Autistic Children Difficult to Find or Access

Despite the increased risk of drowning among autistic children and water safety risks impacting activity and travel planning, parents generally reported *insufficient water safety information* available to them. Parents recalled receiving water safety messages from social media, news, billboards, and personal experiences. One parent pointed out that the information received contributed to a sense of fear without offering actionable recommendations. “It’s so scary to see all those things but the media doesn’t tell you what [to do] for the prevention” (I,30). Parents pointed out that healthcare environments are a great way to reach parents of autistic children. “Going to the doctors, the physical therapist - going to those groups with information is very helpful. That’s where a lot of our population lives and we’re getting information” (VI,126). However, no participants reported receiving significant advice around water safety from their pediatrician or other health professionals.

Parents generally reported *difficulty identifying specialized swimming lessons* that would work for their child. The most commonly reported method of discovery was communication from other parents, whether through online forums or personal references. Once lessons are identified, it can be difficult to know whether it will work for their child. As one parent pointed out, “[It is] hard to vet…these places and look at these people and determine their experience level with what you need them for” (VI,126). Some parents reported that social media is a common way of finding referrals to swimming instruction that has worked well for other families.

There were many barriers parents encountered when trying to enroll their children in swimming lessons. Parents generally noted the *limited availability of specialized or adaptive lessons*, often due to the limited availability of specialized instructors and long waiting lists. One parent expressed frustration at the scarcity of specially trained instructors. “There is a long list. Like for me it took one year to get into the waiting list where I take my kiddo for swimming” (I,30). Many parents also cited *distance to specialized lessons* and *higher costs* as major barriers. Some parents pointed out the benefit of having a reduced price for special needs kids. “That’s why we were going there…it would be cool for insurance to be able to cover this kind of expense for swimming lessons…usually kids [with] autism, they can’t go to group activities” (V,10). Parents sometimes found creative ways to access affordable, specialized swimming lessons. Multiple participants reported taking lessons in Mexico when visiting family. Most parents indicated that *competing priorities*, such as tight schedules filled with therapy sessions, medical appointments, and siblings’ activities increased the difficulty scheduling swimming lessons. “All the therapies, they take many hours…so fitting in water safety lessons…it’s just not as accessible as it should be” (III,7).

Some parents pointed out *racial and ethnic disparities* in swimming lessons and pool access, both geographically and generationally. “Black children are…actively discouraged from swimming. They are not taught to swim. Nobody cares if they know how to swim and I’m sure it’s even worse if you have autism or any of the traits that can come with autism…if it’s harder to teach you how to swim…it’s gonna be considered behavioral instead of developmental” (VI,126). Another parent pointed out that there is a need to have more water safety outreach in Hispanic communities because so many of the parents don’t know how to swim (I,27).

It was common for parents to report *prior negative swimming instruction experiences* before either finding a program that worked or giving up. “We tried twice and I just wait[ed] because there was no progress and he was upset….he was more scared than before…there’s no trained personnel there” (I,27). “Basically, when they see how difficult the kid really is, it’s kind of clear that this isn’t gonna work out in any way shape or form. They don’t have the ability to adapt and modify to what your specific need is” (VI,35).

### Theme 4: Characteristics of Autism Affect Participation in Swimming Lessons and Other Aquatic Activities

When parents overcome barriers and successfully access swimming lessons, they report that challenges may still arise due to common characteristics of autism. The many steps of *preparing for water activities* can be challenging for autistic children. The child will need to “use the restroom, which is sensory averse, and then you have to change, and then you have to get into this pool…and once you’re done, it’s all very sensory averse because now you have to do that inverse” (VI,35). Several parents pointed out that *transitions* from one activity to another, or one phase of an activity to another, can be difficult for their child. “A lot of the lessons are designed for half an hour because that’s how much stamina the kids have but almost, in our case, our kid, he takes 20 minutes to warm up [to] getting into the pool. So you get a ten-minute lesson” (VI,35).

*Sensory overload in pool environments* may be an issue for autistic children, due to noise, lights, chlorine odors, and other sensory stimuli. One parent recalled, “[The indoor pool] space was really echoey…the sensory experience was completely overwhelming and so [he] couldn’t even finish the first lesson” (II,24). Another parent described the need for a “magic pool” that is sensory friendly; “There are not pools without children who are screaming in happiness, and that throws him off” (VI,35).

*Water can be a negative sensory stimulus*. A parent explained how her son’s sensory experience made him fearful of being underwater: “One of the quietest places you can be is…underwater…but the fear point is so much that you can’t put your head underwater…[it] doesn’t translate the way you think it should because we don’t understand that sensory profile” (VI,126). Autistic children often have *strong responses to water recreation gear*. Wearing a life jacket, for some children was reported to be a very negative sensory experience (I,27), while for others it was quite positive. “My daughter’s fine with a life jacket and I think she actually likes the tightness of it” (II,45). One parent described how her son insists on wearing goggles (II,24), while another described how her son “doesn’t like anything [like goggles] on his head or face” (III,7). Another common thread was that *water can also have a positive sensory effect*. As one parent explained, “the water [is] like a weighted blanket…it’s like a massive hug, and that is very calming” (IV,65). This sensory input can have a regulating effect on their child. As another parent pointed out, water is his son’s “happy place, and it’s a good grounding activity…will just totally turn his day around” (IV,19).

Several parents called attention to their child’s *inability to assess how they are feeling* or when they have reached a tipping point. As one parent pointed out, “He doesn’t feel that he’s tired, right, so he can swim for a long time and at some point he…will continue and he will not [realize that he doesn’t] have enough energy anymore to stay afloat” (V,10). Autistic children, some parents pointed out, may also engage in *masking*, an attempt to appear neurotypical. Masking can be exhausting for a child and it can make it difficult for an instructor to understand or assess the child’s needs. As another parent pointed out, her son “is a very good masker. So when he presents, you don’t think that he’s gonna have as much trouble as he then does” (VI,126).

For some children, *inflexibility*,* fixations*,* and obsessive rule-following* can affect their ability to participate in water recreation or swimming lessons. “He likes enforcing rules, especially on other people” (II,24). Another remarked, “He’s really big on safety– his definition of safety” (VI,126). Many of the parents disclosed that their child has *additional diagnoses*, such as ADHD or seizures, and that their children have high medical or psychological needs. As one parent explained, this can impact the child’s ability to participate in regular pool environments. “He gets overstimulated very easily… he has ADHD too. So if he’s trying to pay attention and kids are splashing and doing what kids do in a pool, it just really doesn’t work out” (IV,19).

### Theme 5: Autistic Children Have Unique Swimming Lesson Needs

Parents reported that swimming lessons need to *adapt to communication and learning challenges*. A parent noted of her son: “He doesn’t have any ability to follow directions, even one-step directions. So letting him know or communicating with him the way a typical child would listen at his age, just doesn’t work at all” (IV, 43). Others pointed out that expectations of the timeline for skills acquisition need to be adjusted. “He forgets and he needs a refresher every time. He doesn’t retain it. A good program is one that you can keep going back to because…it’s not like they’re gonna graduate and be done with it” (I,23).

Classes need to be at a *developmentally appropriate* level. One parent reported, “The best experience we had was when he was three and they let him go in the younger kid class, which, I don’t know, sounds kind of silly but developmentally, it was more on par with where he was, and his ability to cooperate and pay attention and that sort of thing, and I think that made the most progress” (V,52).

Many parents indicated that their child with autism benefited from small group or individual lessons. Some parents pointed out that their child *learns by example through observing peers*, and that for these kids, a group lesson can be beneficial. A parent reported, “[It] benefited her to be in a group because she works better off of examples, just them telling her wouldn’t have helped, but her seeing the other kids do it worked better for her” (IV,65).

Parents reported positive experiences in *dedicated environments* or events for families with special needs. “It’s a safe place. Everybody understands that this time period is for our kids…there’s not other kids and families that are like, what’s wrong with that kid” (III,7). Parents expressed a need for additional programs specific to neurodivergent needs. “I think [we need] a swimming context…that is centered around the needs of the neurodivergent, and then everybody else can meet you where you are” (II,45).

*Parent involvement* was also reported as important in their child’s swimming instruction. As one parent said, it “would be good for instructors to survey parents before lessons in order to understand [a] child’s particular problems and needs” (IV,63). Another parent described how “parents should be involved in the swimming…and possibly in-water support, I think it depends on the kid” (III,7).

### Theme 6: Instructor Preparedness is Key to Swimming Lesson Success for Autistic Children

Parents pointed out how necessary it is for instructors to have *experience teaching individuals with autism*. As one parent recalled of an adaptive swimming instructor, “ [She] was a special ed teacher, and…she just got it” (II,45). Programs often endeavor to be accommodating and accepting, not realizing that in addition to this, swim teachers need specific knowledge and skills. “You can’t just sign up your child for any local swim class for kids,” one parent said, “it has to be people who are trained and accepting of your child” (I,23). Parents pointed out that swimming instructors should be *knowledgeable about autism*. As one parent articulated, it was helpful to work with “people who are deeply autism aware…one to one…that person who understands how instruction has to happen, how much time it takes” (II,24).

Characteristics of successful instructors of autistic children, as reported by our parents, include patience, perseverance, a set of *therapist skills* that would help them to motivate their child in the way they need. “That will be good if the teacher knows how to manage the behavior stuff…more like [a] therapist” (III,58). Another said, “it would be great to have like an occupational therapist that’s also a swim instructor” (III,7).

## Discussion

This qualitative assessment of water safety experiences revealed that parents’ experience of having a child with autism and navigating water safety can be challenging. Results suggest the interplay of multiple influences on barriers and choices related to water safety. Common characteristics of autism were reported as increasing parents’ safety-related concerns when in or around water, with water-safety fears impacting family life, activities, and travel. Parents reported that specialized water safety and swimming lesson information was hard to find or access and that common characteristics of autism limited their participation in swimming lessons and other aquatic activities. Parents overviewed some swimming lesson needs and emphasized that having educated, prepared instructors would be critical to making swimming lessons possible for their autistic children.

Existing qualitative research on water safety experiences of children with autism identified many similar challenges for families, such as limitations on activities, safety concerns, instructional challenges, and the benefits of swimming (Mische Lawson et al., 2019; Carter & Koch, 2023). It is clear that vast improvements need to be made across the multiple levels of influence in order to protect this vulnerable population better.

Our results suggest that information on drowning risk and prevention should be proactively provided in settings where families frequent, such as pediatric clinics and therapy offices, as well as on social media. Reports from the parents in our focus groups that they could use more helpful drowning prevention information indicate that materials should provide clear, actionable suggestions for navigating different situations in or around water and provide information on specialized swimming lessons in the area.

Additionally, our results indicate a need for more swimming instructors with training to teach children with autism. In the US, Swim Angelfish’s Swim Whisperers^®^ training and The Autism Society’s Safety on the Spectrum(™) Water and Wandering Program are two examples of programming that can increase the number of swimming instructors with the knowledge to teach children with autism (Swim Angelfish, n.d.; Autism Society, 2024). However, there is a lot more progress needed to make swim schools aware of such programs and to coordinate and fund training.

Even when trained swimming instructors are available, financial barriers can be a factor in preventing autistic children from attending swimming lessons. The prevalence of autism diagnosis is higher among lower socioeconomic status families (QuickStats, 2024). Families with autistic children have significantly higher financial burden due to healthcare costs than those with neurotypical children (Zuvekas et al., 2021). Depletion of emotional and financial resources is common among families of children with psychiatric diagnoses, such as autism (Hickingbotham, 2021). Additionally, parents of children with autism who are from underrepresented communities face a variety of additional stressors and barriers (Iadarola et al., 2019). Potentially including swimming lessons as an insurance benefit for children with autism could help overcome financial barriers. Offering swimming lessons for the whole family, including the child with autism, could help with logistics, increase the water competence of parents, and reduce the autistic child’s anxiety around lessons.

This study is limited by the small number of participants included in the focus groups and the residence of participants in a limited geographical area. The vast majority of the focus group participants were female. Research has identified the need to get father’s perspectives (Lobo, 2020). It is unclear if our results would vary with more male representation among our participants. While the racial and ethnic composition of the group did not differ significantly from the demographics of the recruitment area, the small number of participants meant that we had limited input from Black families. With Black families disproportionately affected by drowning, it is critical that their voice be included in water safety research (Abedin et al., 2024). Given that autism resources in general are more scarce in rural communities (Vanegas et al., 2022), future research should examine the accessibility of adaptive water safety resources in these areas. In addition, future research should include examining the intersection of race or ethnicity and autism, and making concerted efforts to include the perspectives of fathers, non-English speakers, and individuals with autism.

## Conclusions

This study contributes to the small existing body of literature on water safety and autism by elucidating challenges that parents face keeping their autistic children safe around water. In light of the heightened risk for drowning (Guan & Li, 2017), water safety needs to be elevated in importance for families of autistic children. Insights from parents in this study can inform efforts to improve water competency and swimming lesson access and delivery, but also indicate the importance of supervision and utilizing barriers to water access. Organizations providing swimming lessons and water safety instruction can take steps to improve access to adaptive lessons by providing instructors with specialized education around working with autistic learners and offering autism-friendly lesson times with reduced environmental stimuli. In addition to availability, barriers to adaptive swimming lessons such as cost and scheduling need to be addressed. Additionally, attempts should be made to find larger scale solutions, such as including water safety in individualized education programs or individual service plans, providing water safety education during medical and therapeutic visits, and including swimming lessons as a health insurance benefit for children with autism or other developmental disabilities.

## Electronic supplementary material

Below is the link to the electronic supplementary material.

Supplementary Material 1

## Data Availability

Data, anonymized transcripts, and codes are available by request to the corresponding author.
