# Supplementary material for: Parent Perspectives on Water Safety for Children with Autism
Source: J Autism Dev Disord. 2025 Apr 16;56(9):3393–402. doi: 10.1007/s10803-025-06819-7 (PMC13427885; doi:10.1007/s10803-025-06819-7)
Supplement: Supplementary file 1 — Supplementary Material 1 [file 10803_2025_6819_MOESM1_ESM.docx]

**Table 3: Quotes from caregivers of autistic children on themes and subthemes related to water safety**

| Subthemes | Representative Quotes |
| --- | --- |
| Theme 1: Characteristics of autism influence water safety risk | |
| Being drawn to water | "My daughter's deep and abiding fascination with deep water...it has a pull over her and she's almost so hyper focused whenever she's near a body of water... you won't know how focused she is until you take your attention away for a split second." (II,45) |
| Poor sense of danger | "He loves water... loves being in the pool or going to the beach, but he has no fear, no understanding that water is dangerous." (III,7) |
| Wandering and elopement | "It's a challenge to keep her with the group. So that's the big problem with swim lessons is she takes off." (II,45) |
| Impulsivity | “Something’s gonna set him off, and he’s gonna become dangerous to other people. He’s gonna chase somebody or he’s gonna run on the side or he’s gonna splash people inappropriately or he’s gonna invade people’s space.” (II,24). |
| Opposition | "He has resistance to the things that would help keep him safe." (II,24) |
| Communication difficulties | "Following directions and comprehending what the instructor is telling them to do is hard." (I,30) |
| Theme 2: Water safety fears influence family life | |
| Emotional toll of vigilance and planning | "There isn't absolutely anything I do in my day that's not impacted by my child’s autism,...but I think the biggest thing for me is other people's misunderstandings about the level of attention and support my child needs...that you can't just tell him [to not do] something and have him not do it.” (IV,43) |
| Water avoidance | “We avoid those situations where it's easy for him to get away and be someplace with water that we wouldn't see him." (I,23) |
| Water access in travel planning | "It would just be too nerve wracking, even visiting family who has a pool is; I don't think I want to stay there. No, it's just too much to do the monitoring around the pool." (II,45) |
| Theme 3: Water safety and swim lesson information for autistic children difficult to find or access | |
| Insufficient water safety information | "It’s so scary to see all those things but the media doesn’t tell you what for the prevention." (I,30) |
| Difficulty identifying specialized swimming lessons | "Finding in like a regular in a group lessons is easy, but one to one with special needs is really hard.” (I,30) |
| Limited availability of specialized or adaptive lessons | "They're very expensive lessons up north so i prefer [to] go to Mexico and this teacher is [a] specialist with kids with [a] disability.” (III,58) |
| Distance to specialized lessons and higher costs | "There's another swim lesson I heard about for special needs families specifically, but it was [too far] and I couldn't do it…also wildly expensive." (II,24) |
| Competing priorities | "All the therapies, they take many hours...so fitting in, water safety lessons...” (III,7) |
| Racial and ethnic disparities | "Black children are ... actively discouraged from swimming. They are not taught to swim. Nobody cares if they know how to swim and I'm sure it's even worse if you have autism, or any of the traits that can come with autism… if it's harder to teach you how to swim ... It's gonna be considered behavioral instead of developmental" (VI,126) |
| Prior negative swim instruction experiences | “That was an awful experience because they paired her with...just one other kid in the class. The teacher was really nice, but <daughter> still needed one-on-one support and the parent of the other child complained, and yeah, we got kicked out... because the other parent felt like,<daughter> was getting more of the teacher attention then their kid.” (II,45) |
| Theme 4: Characteristics of autism affect participation in swimming lessons and other aquatic activities | |
| Preparing for water activities and transitions | "Transitions are challenging because of the opposition to me and because he can't sense what his body needs, and the spirit of wanting to, the need for dopamine is pretty intense where he has to go find the next adventure, go find the new thing go find the dangerous thing" (II,24) |
|  |  |
| Sensory overload in pool environments | "Had swim lessons at his age inside, the sensory experience was completely overwhelming and so couldn't even finish the first lesson" (II,24) |
| Water can be a negative sensory stimulus | "She didn't move forward because she would not go underwater. She would not like the water in her ears. She did not like the feel, even the thought of it." (IV,63) |
| Strong responses to water recreation gear | "My daughter's fine with a life jacket and I think she actually likes the tightness of it. ... my son would just wear it because it hug you the pressure feels good." (II,45)  "He doesn't like anything [like goggles] on his head or face." (III,7) |
| Water can have a positive sensory effect | "The [bath] water relaxes her." (III,58) |
| Difficulty self-assessing sensations | “She was going to be in the water. It was cold outside. That's no barrier. She'll jump into cold water."(II,45) |
| Masking | "The intensity of his masking. So his only sole goal in the world is to make himself look normal, or neuro-like-typical right, is to be able to cope and so that takes an enormous amount out of him and I didn't understand how much it was drawing down on his battery" (II,45) |
| Inflexibility, fixations, and obsessive rule-following | "He's really big on safety– his definition of safety. " (VI,126) |
| Other diagnoses and physical challenges | "Both of my kids...have a great deal of difficulty imitating; like you can say do this... and they can't imitate movement... So it's really a lot of one-on-one support is needed in the swim lesson." (II,45) |
| Theme 5: Autistic children have unique swimming lesson needs | |
| Communication and learning challenges | "I've really got to talk to him like a storyboard... This is how you have to move this. This is what this looks like and then model it for him. It's really got to be slowed down - someone willing to really slow it down" (V,16) |
| Developmentally appropriate | "He's bigger than anybody else his age... but then socially he is actually much younger and so for lessons also... he might actually do really well with kids who are younger like six seven years old eight years old maybe. But I don't want him to feel out of place when he's in a lesson with them. So it's better for us to just find a small one or two kids in the neighborhood and make sure that we align up and sort of swim together and he feels pulled in their direction because he'll play with them. Yeah, but I can't match up the lesson in the way that those two things feel like they're okay for him." (II,24) |
| Learn by observing peers | "She loves to observe kids. She copies [what the other kids are doing]." (III,29) |
| Dedicated learning environment | "There's a pool...they just close it off just for families with kids with disabilities... it's a safe place. everybody understands that this time period is for our kids...there's not other kids and families that are like “what's wrong with that kid”, or it's just like a safer environment where we're all good to just be ourselves and not have that stigma." (III,7) |
| Parent involvement | “A successful instructor should get to know the child a little bit first to see...where they're at. And to take a lot of direction from parents." (III,7) |
| Theme 6: Instructor preparedness is key to swimming lesson success for autistic children | |
| Experience teaching individuals with autism | "[It is] difficult for traditional swim teachers...it's not left foot- right foot...there's a thousand more steps in between for children with…autism or...different disabilities." (VI,35)  “It was successful because they had a good background and they were expecting certain behaviors and they were very tolerant and again, they were very patient." (I,23) |
| Knowledgeable about autism | “It is important that there are instructors who know how to work with autistic kids, because there are some things that if you know–how to process sensory input, you can change the environment in such a way to make it easier. Or you can ignore that and it will make it infinitely harder.” (interview) |
| Therapist skills | "That will be good if the teacher knows how to manage the behavior stuff.... you know, like probably more like [a] therapist." (III,58) |
